# High performance lithium-sulfur batteries for storing pulsed energy generated by triboelectric nanogenerators

**DOI:** 10.1038/s41598-017-00545-6

**Published:** 2017-03-27

**Authors:** Weixing Song, Chao Wang, Baoheng Gan, Mengmeng Liu, Jianxiong Zhu, Xihui Nan, Ning Chen, Chunwen Sun, Jitao Chen

**Affiliations:** 1 Beijing Institute of Nanoenergy and Nanosystems, Chinese Academy of Sciences, National Center for Nanoscience and Technology (NCNST), Beijing, 100083 P.R. China; 2 Beijing National Laboratory for Molecular Sciences, College of Chemistry and Molecular Engineering, Peking University, Beijing, 100871 P.R. China; 3 School of materials science and Engineering, University of Science and Technology Beijing, Beijing, 100083 P.R. China

## Abstract

Storing pulsed energy harvested by triboelectric nanogenerators (TENGs) from ambient mechanical motion is an important technology for obtaining sustainable, low-cost, and green power. Here, we introduce high-energy-density Li-S batteries with excellent performance for storing pulsed output from TENGs. The sandwich-structured sulfur composites with multi-walled carbon nanotubes and polypyrrole serve as cathode materials that suppress the shuttle effect of polysulfides and thus preserve the structural stability of the cathode during Li-ion insertion and extraction. The charging time and energy storage efficiency of the Li-S batteries are directly affected by the rotation rates of the TENGs. The average storage efficiency of the batteries for pulsed output from TENGs can exceed 80% and even reach 93% at low discharge currents. The Li-S batteries also show excellent rate performance for storing pulsed energy at a high discharge current rate of 5 C. The high storage efficiency and excellent rate capability and cyclability demonstrate the feasibility of storing and exploiting pulsed energy provided by TENGs and the potential of Li-S batteries with high energy storage efficiency for storing pulsed energy harvested by TENGs.

## Introduction

Mechanical energy produced everywhere in daily life has been showing considerable promise as a sustainable power supply^1–5^. Many conversion mechanisms for mechanical energy harvesting, such as electromagnetic effects^6, 7^, piezoelectric effect^3, 8–11^, and electrostatic and triboelectric effects^12–18^, have been well developed during recent decades. Among them, triboelectric nanogenerators (TENGs), which have been rapidly developed in recent years, show excellent potential for applications considering their appealing characteristics including high power density, low fabrication and maintenance costs^19–21^. TENGs work on the coupling between the triboelectric effect and electrostatic induction, and TENGs are the applications of Maxwell’s displacement current in energy and sensors^22^. They have been proved to enable converting mechanical energy from ambient mechanical motions into electrical energy. The mechanical motions cover various daily aspects such as human motion, mechanical triggering, rotating tires, wind, waves, etc.^23^. However, the energy harvested from ambient environments is generally unsustainable and time-dependent, considering the randomness of mechanical energy sources and pulsed alternating current (AC) outputs. In addition, the pulsed output with high voltage and low current cannot directly serve as steady power sources for driving electronics.

Therefore, developing energy storage devices with high storage efficiencies is necessary for exploiting the pulsed outputs from TENGs. Although the power generated from TENGs could be stored in lithium ion batteries or supercapacitors^24–27^, the specific capacity and energy released from these storage devices are still low due to the intrinsic features of the materials of these energy storage devices and poor adaptability of the storage materials to the pulse-type current. The efficient storage of energy from TENGs remains a considerable challenge.

Here, we present an effective cathode material composed of sandwich-structured sulfur composites with multi-walled carbon nanotubes (MWCNTs) and polypyrrole (PPy) for Li-S batteries to meet the energy storage requirement of pulsed output harvested by TENGs. Li-S batteries have been demonstrated as one of the most promising next generation energy storage systems^28–31^. Sulfur cathodes have a high theoretical specific capacity of 1675 mAh/g, which is an order of magnitude higher than that of current insertion cathodes for Li-ion batteries, and have a high energy density of 2600 Wh/kg^28^. This is due to the ability of sulfur atoms to convert elemental sulfur into lithium sulfide (Li_2_S) after accepting two electrons. In addition, the fact that sulfur is naturally abundant, relatively lightweight and an inexpensive product from industry also favor the development and utilization of Li-S battery technologies^29^.

**Figure 1:**
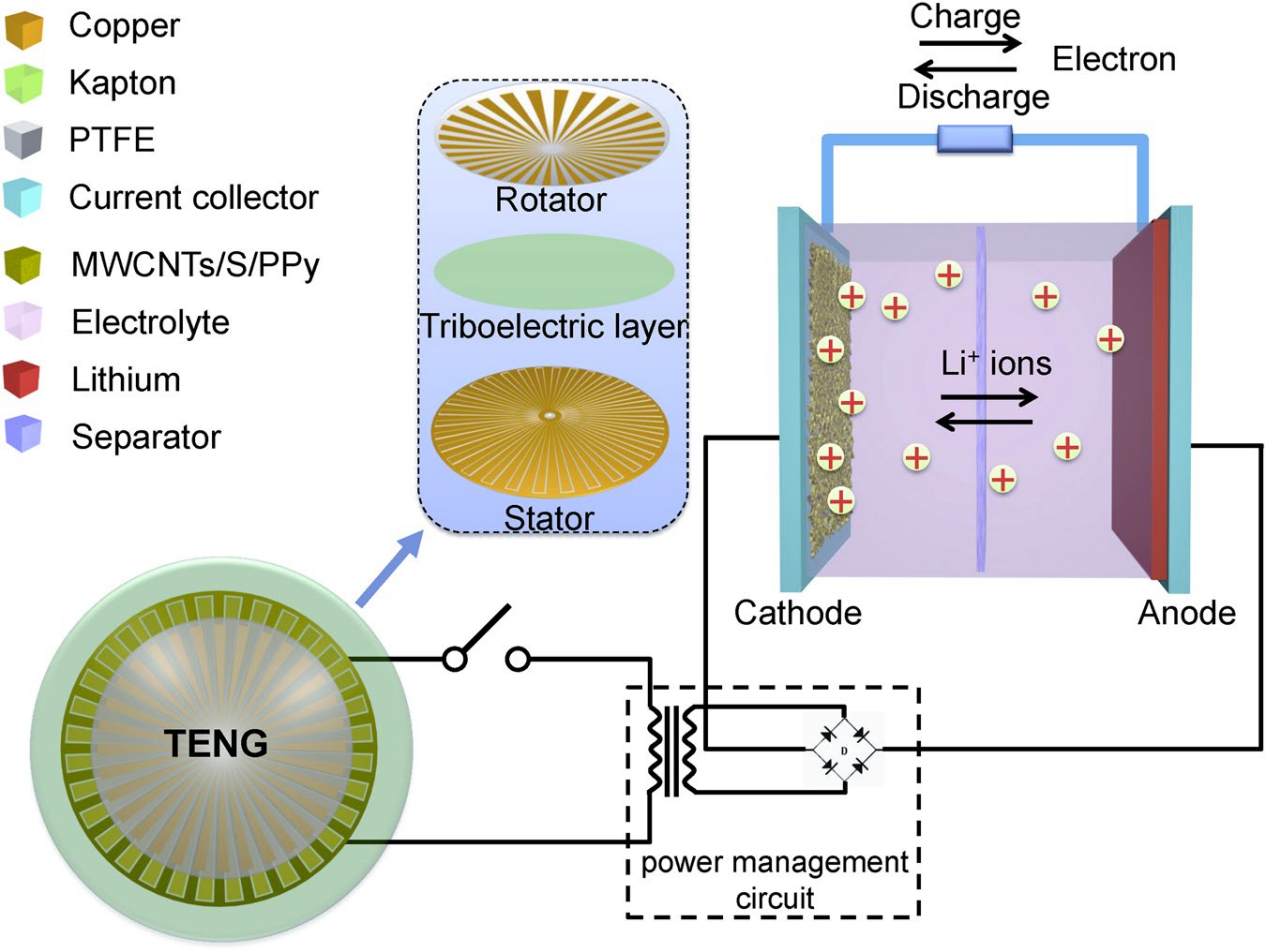
Schematic image of storing the pulse energy generated by the TENGs in a Li-S battery with MWCNTs/S/PPy as the cathode.

In this work, typical pulsed electrical energy generated by radial-arrayed rotary TENGs was efficiently collected and stored using Li-S batteries (Fig. 1). The Li-S battery with a MWCNTs/S/PPy composite as the cathode exhibits excellent pulsed energy storage efficiency of over 80%, which is directly produced from typical radial-arrayed rotary TENGs. The coupling of TENGs with different rotary rates and the corresponding charge-discharge process of the Li/S batteries were examined to investigate the storage capacity and cyclic stability of Li/S batteries specifically for pulsed power. After being charged by the TENGs, the batteries show a high average specific capacity of 1005 mAh/g. The excellent cyclic stability of Li-S batteries charged by TENGs 300 times indicated the feasibility of storing energy harvested from TENGs. These results demonstrate that a TENG coupled with such Li-S batteries is a viable energy generation and collection technology for the integration of intermittent energy from the environment into grids.

## Results and Discussion

### Characteristics of the Li-S batteries with the MWCNTs/S/PPy composite

**Figure 2:**
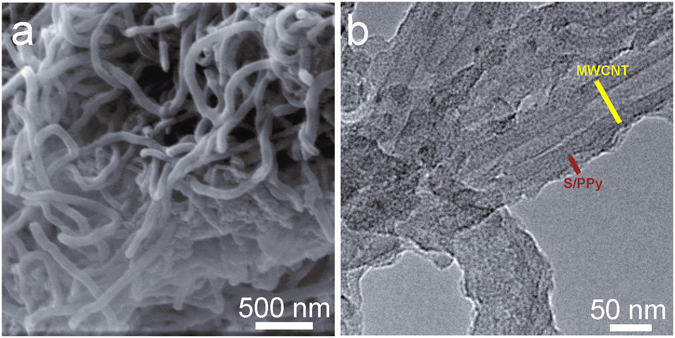
(**a**) SEM and (**b**) TEM images of the MWCNTs/S/PPy composites.

The cathode materials of Li-S batteries are composed of MWCNTs/S/PPy composites, which were prepared by depositing S nanoparticles and successively polymerizing PPy on the MWCNT frameworks *in situ* (Fig. 2a)^32^. The S nanoparticles and PPy were uniformly coated on the surface of the MWCNTs as shown in Fig. 2b and Figure S1. The TEM image (Fig. 2b) also shows a uniform tubular shape with internal void space. Due to the specific structure, the sulfur was successfully restricted in the sandwich interlayer. Moreover, MWCNTs not only serve as a conductive network for sulfur, they also absorb some polysulfide molecules and therefore help to suppress the shuttle effect of polysulfides. The shuttle effect has been considered to be mainly responsible for the capacity fading in Li-S batteries because polysulfides prefer to dissolve into the electrolyte^33^. Meanwhile, PPy is expected to further improve the conductivity and simultaneously maintain the dual structure of the MWCNTs/S/PPy composite, thereby helping to restrain the spreading of the polysulfide.

**Figure 3:**
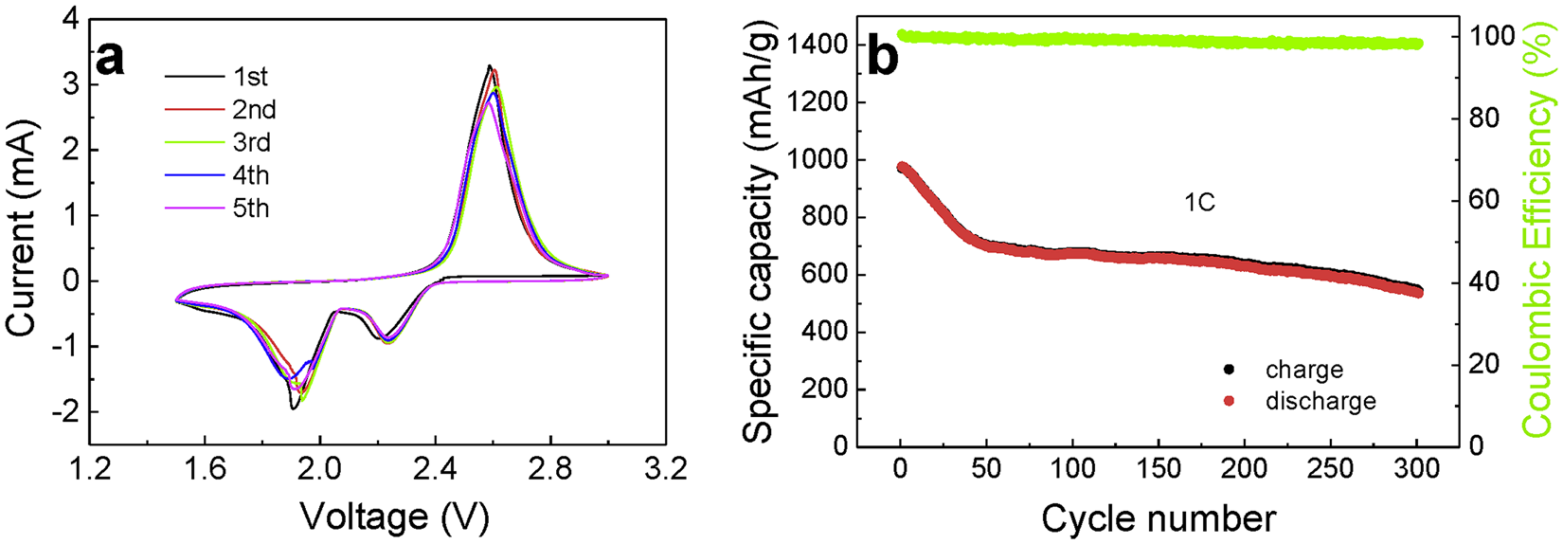
(**a**) Cyclic voltammograms of the MWCNTs/S/PPy composite electrode in the voltage range of 1.5~3 V vs. Li^+^/Li, recorded at a potential scanning rate of 0.5 mV. (**b**) Cycling performance of the MWCNTs/S/PPy composite at a current density of 1 C.

To investigate the effects of rational design and the synergetic effect of the MWCNTs/S/PPy framework, the electrochemical behavior of the MWCNTs/S/PPy composites as the cathode for Li-S batteries was explored using cyclic voltammetry (CV). Figure 3a shows the CV curves of the sulfur composite electrode during the first 5 cycles. Two cathodic peaks are observed due to the multiple reduction of sulfur in the presence of Li^+^ ions. The peak at 2.2 V is related to the reduction of S_8_ to long chain lithium PS (Li_2_S_*x*_, 4 < *x* < 8). The next peak at about 1.9 V is associated with further reduction of the long chain PS to Li_2_S_2_ or Li_2_S. In the subsequent anodic scan, only one anodic peak at about 2.5–2.6 V is observed, which can be attributed to the oxidation of Li_2_S_2_ and Li_2_S to Li_2_S_8_. All of the successive CV cycles barely change, indicating the good durability of the sulfur composite. Figure 3b shows the cyclability of the sulfur composite at a current density of 1 C (1 C = 1675 mA/g). The MWCNTs/S/PPy composites exhibited an initial specific discharge capacity of 977 mAh/g. The capacity fading rate is relatively fast over the first 40 cycles. However, the subsequent capacity fading rate is only 0.1% per cycle. After 300 cycles, the capacity still remains at 540 mAh/g. The coulombic efficiency is near 100% during cycling, indicating that the sulfur shuttle effect is diminished to a very low level.

### Performance of the TENG

**Figure 4:**
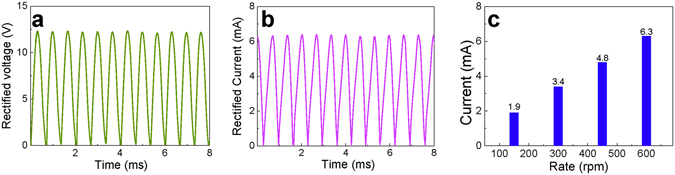
(**a**) Output voltage and (**b**) current of the TENGs. (**c**) Dependence of the amplitude of the output short circuit current on the rotation rate of the TENGs.

The radial-arrayed rotary TENGs can sustainably produce pulsed output and are used to charge the Li-S batteries. A typical rotary TENG is composed of a rotator, a stator and a triboelectric layer consisting of 100 μm of Kapton attached on the stator. It has a radial-arrayed pattern on two circular planes (Fig. 1). The surface of the stator consists of two delicately designed complementary-patterned electrodes with gaps between two radial lines. The rotation of the rotor can evenly and continuously produce alternating triboelectric potential and current, which behaves like an AC output between electrodes (Figure S2), relying on the coupling between the triboelectric effect and electrostatic induction^34^. By introducing a power management circuit including a transformer and a rectifier (Fig. 1), the pulsed ACs can be reasonably adjusted to yield adequate direct voltage and current, as shown in Fig. 4a and b. A TENG produces an open-circuit voltage (*V*_oc_) of about 12.2 V and a short-circuit current (*I*_sc_) of 6.3 mA at a rotation rate of 600 rpm, which corresponds to a frequency of 600 Hz when the central angle of the rotator unit is 3°. The electric output of the TENGs is directly concerned with the rotation rates of the rotors. The amplitude of *I*_sc_ linearly increases as the rotation rate increases (Fig. 4c), while the amplitude of V_oc_ is based on the nature of the materials and the relative position between the rotator and the stator^34^. The output current functions as an AC, and therefore, the average value of the rectified current *I*_*a*_ can be calculated according to the AC mode as

1 $${I}_{a}=2/\pi \cdot {I}_{{\rm{\max }}}$$

where *I*_max_ is the peak value of the output current.

### Charging the Li-S batteries by the TENG

**Figure 5:**
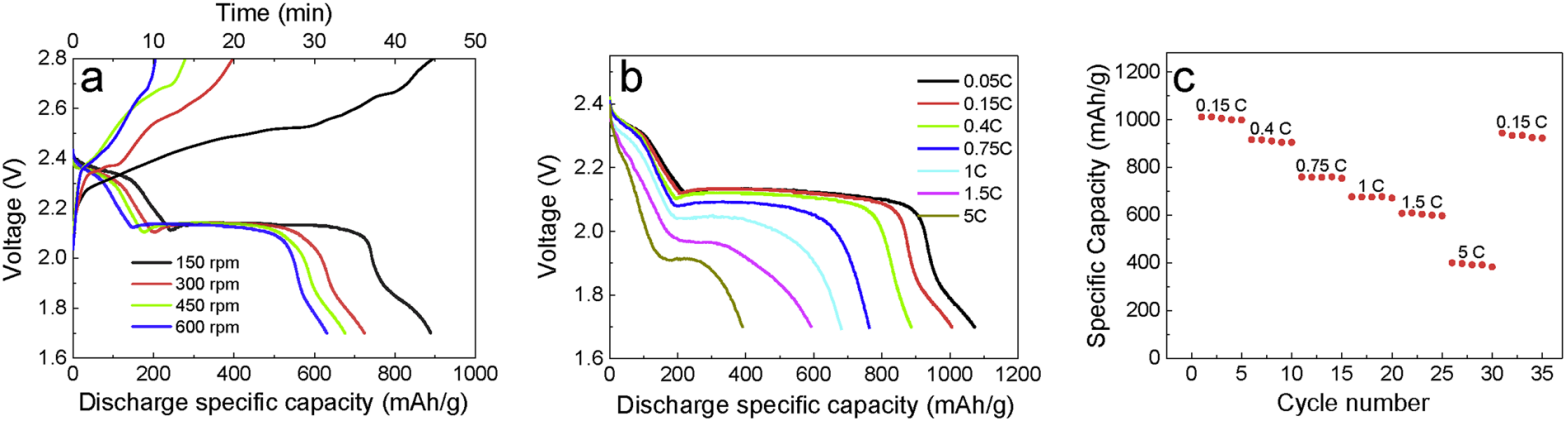
(**a**) Charge profiles of the MWCNTs/S/PPy composites from the TENGs at different rotating rates and discharge profiles at 0.5 C. (**b**) Discharge profiles of the composites charged by the TENGs at 150 rpm at different current rates. (**c**) Rate performance of the composite electrode with current rates ranging from 0.15 C to 5 C.

When the Li-S batteries were charged by the rotary TENG, the rotation rate of the TENG directly influences and determines the charging time of the batteries due to the different generated current values. The TENG generates an average current of about 6.2 mA and can quickly charge the batteries to 2.8 V in 11 min at a rotation rate of 600 rpm (Figs 4c and 5a). The decreasing rate would result in lower currents and increasing charge time. However, the discharge specific capacity presents the opposite trend: the battery charged at a slower rate has a higher capacity, as shown in Fig. 5a. When charging at a rotation rate of 150 rpm, the battery has a discharge specific capacity of 869 mAh/g at a discharging current of 0.5 C. Even charging at 600 rpm, the capacity still remains greater than 600 mAh/g, which is much higher than the value of 100–200 mAh/g in common lithium ion batteries. From the charge/discharge profile, one typical charge voltage plateau and two discharge voltage plateaus are observed for the lithium-sulfur batteries, which agree well with the results of the cyclic voltammetry shown in Fig. 3a. At different charge rotation rates of the TENG, the small changes in the charge plateau indicate slight polarization of the cathode materials, which indicates that the MWCNTs/S/PPy composite can adapt well and store the pulsed energy from TENGs.

Using the same charge rotation rate of 150 rpm, the discharge profiles at different current densities from 0.05 C to 5 C are shown in Fig. 5b. The MWCNTs/S/PPy composite electrode delivers a high specific capacity of 1080 mAh/g at a current density of 0.05 C (83.75 mA/g), which is comparable to some previously reported results for lithium-sulfur battery anodes with high volumetric energy^29, 35^. When the discharge current density increases to 1 C (1675 mA/g) and 5 C (8375 mA/g), the composite electrode still shows high specific capacities of 675 mAh/g and 395 mAh/g respectively, which are equivalent to the values of batteries charged using DC^36^. The rate performance was investigated at various cycling rates. Figure 5c shows that the composite has an excellent rate performance. The discharge capacities of the composite electrode reach about 1005, 910, 758, 675, 605, and 395 mAh/g at rates of 0.15 C, 0.4 C, 0.75 C, 1 C, 1.5 C, and 5 C, respectively. Furthermore, the discharge capacity can be recovered to 930 mAh/g at 0.15 C after cycling, indicating the excellent reversibility of the MWCNTs/S/PPy composite.

The energy storage efficiency (*η*) of the batteries is an important parameter for the utilization of the energy collected from the TENGs. The charge energy is calculated by integrating the area of the charge *V*-*t* curves and then multiplying the average value of the current *I*_a_ in Fig. 5a,

2 $${E}_{{\rm{charge}}}={I}_{a}{\int }_{0}^{t}V(t)dt$$

The discharge energy is also calculated by integrating the area of the discharge curves in Fig. 5a,

3 $${E}_{{\rm{discharge}}}={\int }_{0}^{C}V(C)dC$$

where *V* is the voltage of the batteries and *C* is the discharge specific capacity. Therefore, the storage efficiency can be measured as

4 $$\eta ={E}_{{\rm{discharge}}}/{E}_{{\rm{charge}}}$$

**Figure 6:**
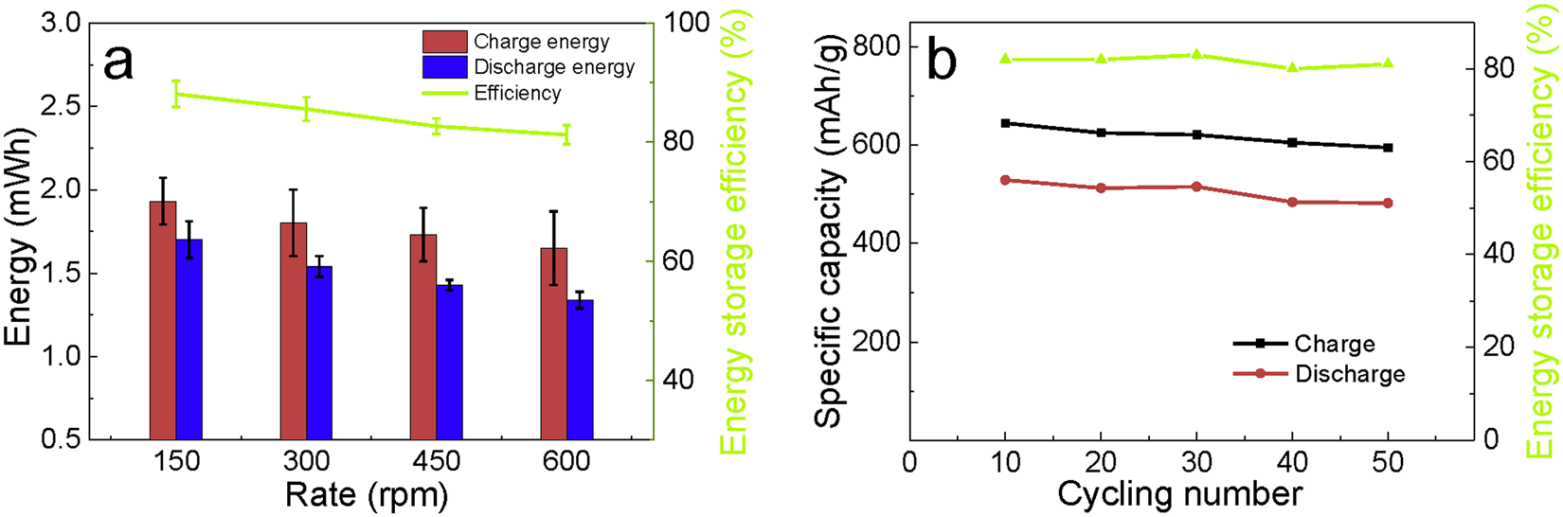
(**a**) Average charge energies of the lithium-sulfur batteries charged by the TENGs at different rotating rates, average discharging energies of the batteries at 0.5 C, and a comparison of the average energy storage efficiencies of the batteries at different rotating rates. Each charge-discharge process is tested for 20 cycles to obtain the average value. (**b**) Cycling performance of the MWCNTs/S/PPy composite at the charge rotating rate of 300 rpm of TENG and the discharge current density of 1 C.

The charge process of batteries was performed at different rotation rates from 150 rpm to 600 rpm, and each test was repeated for 20 cycles in order to obtain the average charge energy, discharge energy and storage efficiency. The average storage efficiency can reach about 84% at the discharge rate of 0.5 C when the rotation rate of the TENGs is decreased to 150 rpm (Fig. 6a). Higher rotation rates lead to a slight decline, which can be ascribed to more polarization in the cathode induced by the increasing current density. When the batteries discharge at a current density of 0.05 C, the average efficiency can reach 93%. This measurement demonstrates that the pulsed output energy of the TENGs can be efficiently stored in Li-S batteries, and the coupled TENG-battery system is feasible as a high power source. In addition, due to the promising property of harnessing various ambient motions, the integration of TENGs with such Li-S batteries can be expected to provide sustainable and stable energy. We further study the cycling performance of the MWCNTs/S/PPy composite at the charge rotating rate of 300 rpm and the discharge current density of 1 C. The average charge and discharge specific capacities and energy storage efficiency for each 10 cycles are calculated and shown in Fig. 6b. During 50 cycles the specific capacities slightly decline with the increase of the cycle number, but the storage efficiency exhibit a rather steady trend, which indicates that the batteries have good cycling performance for storing the pulsed energy from TENG.

## Conclusion

The pulsed output current generated by TENGs can be efficiently stored in Li-S batteries with the MWCNTs/S/PPy composite as the cathode. The discharge specific capacity can exceed 1000 mAh/g at current densities lower than 0.15 C. The energy storage efficiency of the Li-S batteries for pulsed energy from TENGs can exceed 80%, and an increasing rotation rate reduces the charge time despite the slight loss of storage efficiency. The excellent rate performance of such Li-S batteries for storing energy harvested by TENGs indicates that the MWCNTs/S/PPy composite has a remarkable adaptability for storing the pulse energy from TENGs.

## Experimental Section

### Fabrication of the TENG

A TENG consists of a rotator and a stator coated with a polymer film, as shown in Fig. 1a. The patterns are fabricated using print circuit board technology^34^.

### Fabrication of Li-S batteries

The MWCNTs/S/PPy composites were synthesized according to a previous report^32^. Briefly, MWCNTs were dispersed in a 1% sodium dodecyl sulfate (SDS) solution in 100 mL of water, followed by adding 8 g of Na_2_S_2_O_3_ and stirring for 10 min. Subsequently, 13 g of HCl was slowly added to the mixture, and then, this mixture was stirred for 1 h, which produces sulfur on the MWCNTs. Prior to adding pyrrole, sodium p-toluenesulfonate (BDSNa) was added to the mixture and stirred for 10 min at 0 °C. FeCl_3_ was added and stirred overnight at 0 °C. Finally, the composite power was washed several times with water via vacuum filtration and dried at 60 °C for 12 h. The sulfur-based composites were first mixed with acetylene black and polyvinylidene fluoride (PVDF) binder in a weight ratio of 7:2:1 in N-methyl-pyrrolidone. The mixture was uniformly spread on an Al foil with a doctor blade. CR2032 coin cells were assembled using lithium foil as the anode and lithium bis-trifluoromethanesulfonylimide (LiTFSI, 0.6 M in DOL/DME) with 0.4 mol/L LiNO_3_ as the electrolyte.

### Characterization

Electron microscopy imaging was performed by scanning electron microscopy (SEM, SU8020, Hitachi, Japan) and an FEI F20 transmission electron microscope (TEM, F20, FEI, USA). The output current and voltage of the TENGs were measured using a SR570 low-noise current amplifier (Stanford Research System) and an oscillometer (Lecory HDO6104), respectively. The galvanostatic cycling and discharging at different currents were carried out using a battery tester (LAND CT2001A) from 1.7 to 2.8 V versus Li^+^/Li. During charging, the Li/S batteries, a power management circuit including a transformer and a bridge rectifier were used, and the voltages of the cells were recorded by the Keithley 6514. The specific capacity values were calculated based on the mass of sulfur in the samples determined by thermogravimetric analysis. The loading of the active materials was around 1 mg/cm^2^. Cyclic voltammetry was performed on an electrochemical workstation (Autolab PG302N) at a scan rate of 0.05 mV/s in the voltage range of 1.5~3 V versus Li^+^/Li.

## Electronic supplementary material

Supporting information
