# Supplementary material for: High performance lithium-sulfur batteries for storing pulsed energy generated by triboelectric nanogenerators
Source: Sci Rep. 2017 Mar 27;7:425. doi: 10.1038/s41598-017-00545-6 (PMC5428700; doi:10.1038/s41598-017-00545-6)
Supplement: Supplementary file 1 — Supporting information [file 41598_2017_545_MOESM1_ESM.pdf]

Supporting Information for

High performance lithium-sulfur batteries for storing pulsed energy generated by triboelectric nanogenerators

Weixing Song[1,*], Chao Wang[2], Baoheng Gan[1,3], Mengmeng Liu[1], Jianxiong Zhu[1], Xihui Nan[1], Ning Chen[3], Chunwen Sun[1,*], Jitao Chen[2,*]

[1]Beijing Institute of Nanoenergy and Nanosystems, Chinese Academy of Sciences; National Center for Nanoscience and Technology (NCNST), Beijing 100083, P. R. China

[2]Beijing National Laboratory for Molecular Sciences,College of Chemistry and Molecular Engineering, Peking University Beijing 100871, P. R. China

[3]School of materials science and Engineering, University of Science and Technology Beijing, Beijing 100083, P. R. China

*songweixing@binn.cas.cn, sunchunwen@binn.cas.cn, chenjitao@pku.edu.cn

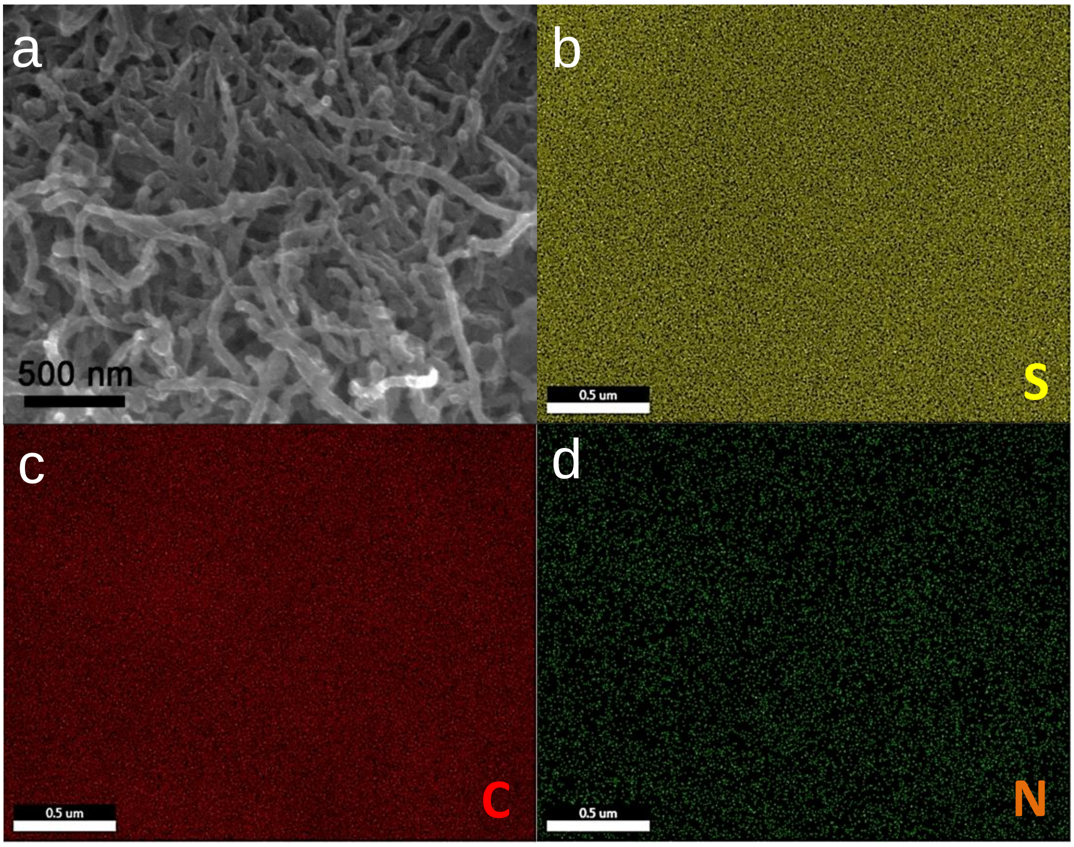

Figure S1. (a) SEM image of the MWCNTs/S/PPy composite. (b) Corresponding energy dispersive X-ray mapping of (b) sulfur, (c) carbon, and (d) nitrogen.

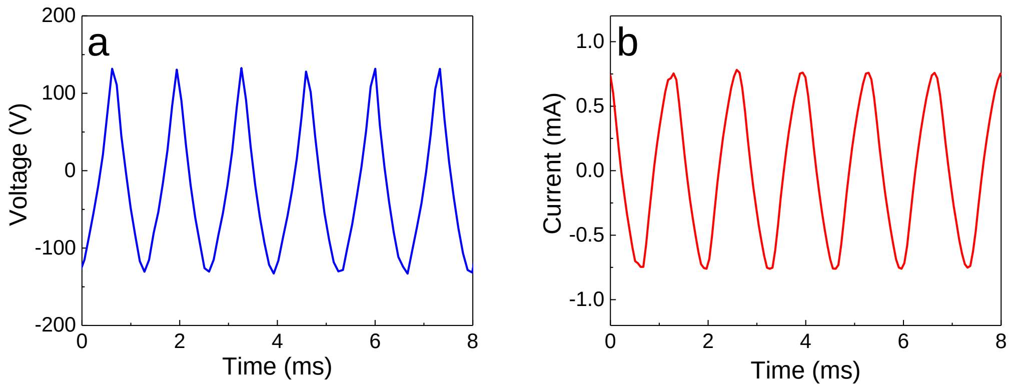

Figure S2. (a) Output voltage and (b) current of the rotary TENGs.
